# 

**Published:** 1915-06-13

**Authors:** 


### THE HOSPITAL

The Modern Newspaper of Administrative Medicine and Institutional Life.

No. 1513. Vol. LVIII. Sunday, June 13, 1915. [REGISTERED AS A NEWSPAPER] PRICE ONE PENNY

